# Clinical implementation of AXB from AAA for breast: Plan quality and subvolume analysis

**DOI:** 10.1002/acm2.12329

**Published:** 2018-04-25

**Authors:** Alexandra Guebert, Leigh Conroy, Sarah Weppler, Majed Alghamdi, Jessica Conway, Lindsay Harper, Tien Phan, Ivo A. Olivotto, Wendy L. Smith, Sarah Quirk

**Affiliations:** ^1^ Department of Physics and Astronomy University of Calgary Calgary AB Canada; ^2^ Division of Medical Physics Tom Baker Cancer Centre Calgary AB Canada; ^3^ Division of Radiation Oncology Department of Oncology University of Calgary Calgary AB Canada; ^4^ Department of Oncology Tom Baker Cancer Centre Calgary AB Canada

**Keywords:** Acuros, breast, clinical implementation, dose calculation algorithms

## Abstract

**Purpose:**

Two dose calculation algorithms are available in Varian Eclipse software: Anisotropic Analytical Algorithm (AAA) and Acuros External Beam (AXB). Many Varian Eclipse‐based centers have access to AXB; however, a thorough understanding of how it will affect plan characteristics and, subsequently, clinical practice is necessary prior to implementation. We characterized the difference in breast plan quality between AXB and AAA for dissemination to clinicians during implementation.

**Methods:**

Locoregional irradiation plans were created with AAA for 30 breast cancer patients with a prescription dose of 50 Gy to the breast and 45 Gy to the regional node, in 25 fractions. The internal mammary chain (IMC_CTV_) nodes were covered by 80% of the breast dose. AXB, both dose‐to‐water and dose‐to‐medium reporting, was used to recalculate plans while maintaining constant monitor units. Target coverage and organ‐at‐risk doses were compared between the two algorithms using dose–volume parameters. An analysis to assess location‐specific changes was performed by dividing the breast into nine subvolumes in the superior–inferior and left–right directions.

**Results:**

There were minimal differences found between the AXB and AAA calculated plans. The median difference between AXB and AAA for breast_CTV_ *V*_95%_, was <2.5%. For IMC_CTV_, the median differences *V*_95%_, and *V*_80%_ were <5% and 0%, respectively; indicating IMC_CTV_ coverage only decreased when marginally covered. Mean superficial dose increased by a median of 3.2 Gy. In the subvolume analysis, the medial subvolumes were “hotter” when recalculated with AXB and the lateral subvolumes “cooler” with AXB; however, all differences were within 2 Gy.

**Conclusion:**

We observed minimal difference in magnitude and spatial distribution of dose when comparing the two algorithms. The largest observable differences occurred in superficial dose regions. Therefore, clinical implementation of AXB from AAA for breast radiotherapy is not expected to result in changes in clinical practice for prescribing or planning breast radiotherapy.

## 1 INTRODUCTION

### 1.A Motivation

There are currently two dose calculation algorithms available in Varian Eclipse software (Varian Medical Systems, Palo Alto, USA): Anisotropic Analytical Algorithm (AAA) and Acuros External Beam (AXB). AXB was introduced in 2010 to address limitations of AAA in inhomogeneous regions, including overestimation of dose in the lung,^1^, ^2^, ^3^, ^4^ and the over/under estimation of dose beyond low/high density materials.^5^, ^6^ While many centers with Varian Eclipse software have access to AXB, implementation of a new dose calculation algorithm requires a thorough understanding of how this change will affect plan quality and, subsequently, clinical practice. This paper characterizes differences in dose–volume parameters and plan quality when implementing AXB from AAA for breast cancer radiotherapy planning.

### 1.B Background

AXB is based on solving the Linear Boltzmann Transport Equation numerically, and calculates dose to the medium instead of the dose to water, resulting in more accurate calculation for inhomogeneous tissues than AAA. In breast radiotherapy, there is a large range of tissue densities in the irradiated volume (breast, lung, bone, air). The irradiation of internal mammary chain (IMC) nodes using a modified wide tangent field is growing in popularity,^7^ and this technique results in a larger volume of lung included in the field than standard tangents. This may introduce larger uncertainties in calculated dose due to inhomogeneous interfaces.

Fogliata et al. characterized the performance of AXB for simple two‐tangent field plans in different breast tissue types (adipose vs. ductal), and for lung inside and outside the radiation field.^8^ This investigation determined that AXB could differentiate between breast tissue types and suggested that this may improve accuracy of dose calculation for patient treatments. This study also showed that for lung dose calculations the largest difference between AXB and AAA was for deep inspiration breath hold (DIBH) with consistent overestimation by AAA.^8^

Our institution currently uses AAA for breast radiotherapy planning, and this study was designed to inform the clinical implementation of AXB for four‐field breast planning in our clinic. Specifically, we aim to determine if a clinically relevant difference exists between the two calculation algorithms in this context. For this purpose, we define a clinically relevant difference as a difference in the appearance of the AXB plans (through evaluation of isodose lines or DVH curves) when compared to AAA plans, that may be noticeable or concerning to a physician reviewing the plan. The largest differences between dose calculations by AXB and AAA occur in patients planned during DIBH as lung density is reduced through this maneuver.^8^ Both breast and IMC_CTV_ are assessed for coverage and plan quality; in addition, the impact on relevant organs at risk is evaluated.

## 2 METHODS

### 2.A Patient characteristics, volume definitions, and dose prescription

This study used the DIBH CT datasets from 30 patients treated with radiation following breast conserving surgery. Patients were scanned in the supine position, immobilized on a wing board with both arms over the head, with CT slice thickness of 2 mm (Philips Big Bore, Philips, Massachusetts). The right breast, lungs, heart, and IMCs were contoured according to ESTRO guidelines.^9^ Specifically, the IMC CTV (IMC_CTV_) was defined as a 5 mm expansion around the medial vascular structure from the first to third interspaces. Treatment plans were developed on this dataset using clinical field borders, and the breast was contoured for dose evaluation purposes only. The superficial region was defined as a 5 mm margin deep to the external body contour bordering on, but excluding, the breast. This represents the dose in the superficial region but is not anatomically representative of the skin.^10^ A summary of patient characteristics can be found in Table 1. The prescription dose for the whole breast was 50 Gy in 25 fractions and the mid‐axillary dose was 45 Gy in 25 fractions. The IMC_CTV_ was covered by 80% of the tangent dose.

**Table 1:** Patient characteristics

	Median	Range
Breast volume (cc)	601.1	102.4–1298.9
Lung volume (cc)	2446.4	1285.5–3147.1
Mean HU across largest separation	−454.6	−597.5 to −327.5
Path length at field edge (cm)	24.7	15.1–32.5

### 2.B Separation across beam path

**Figure 1:**
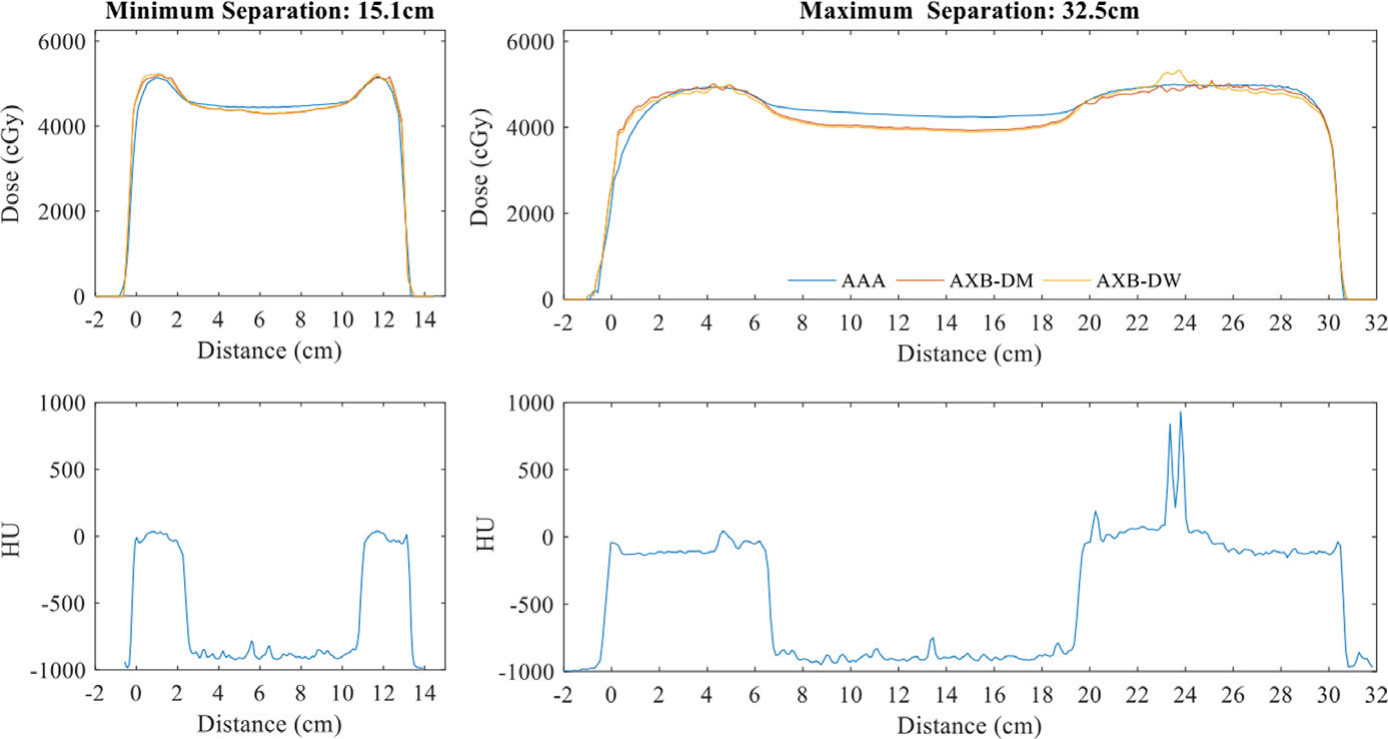
Separation profiles across field edge for patient with (a) smallest and (b) largest separation. The bottom plots show the CT profile along the same line. The 0 distance corresponds to the medial breast.

The beam path length along the 2500 cGy isodose line ranged from 15.1 to 32.5 cm. The profiles of the total plan dose, as well as the CT profile along the line, for AXB and AAA are provided in Fig. 1.

### 2.C Treatment planning and dose calculation algorithms

Forward‐planned right breast field‐in‐field plans were created for each patient by the same certified dosimetrist. All patients were planned with a four‐field, monoisocentric technique. Wide tangent beams were defined by anatomical landmarks to include the breast and IMCs and the supraclavicular and axillary nodes were covered with a parallel opposed anterior–posterior pair with the anterior field angled away from the spine if required. Energy, segments, and beam weightings were employed to improve dose homogeneity. A combination of 6 and 15 MV beams were used for the tangents. 13/30 (43%) of patients were planned with only 6 MV beams, 6/30 (20%) with only 15 MV beams, and 11/30 (37%) with a combination of 6 and 15 MV beams. A half‐beam block technique was used to minimize divergence into the lung with jaws closed to the central axis.

The dose distribution for each patient treatment plan was computed with: AAA [Anisotropic Analytical Algorithm (Version 11.0.31)] and AXB [Acuros External Beam (Version 11.0.31)]. Both algorithms were implemented in Eclipse version 11 treatment planning system (Varian Medical System). Plans were optimized with AAA and then recalculated using AXB dose‐to‐medium (AXB‐DM) and AXB dose‐to‐water (AXB‐DW) while keeping the field geometry, energy, beam segments, prescription point, and number of Monitor Units (MU) constant. The calculation grid for both algorithms was 0.25 cm. All calculations were performed with heterogeneity corrections turned on.

### 2.D Plan Comparison and Evaluation Metrics

Dose–volume histogram (DVH) parameters were retrieved using an in‐house MATLAB R2016a (The MathWorks, Inc., Natick, MA) Graphical User Interface (GUI) with a bin size of 0.1 cGy. DVH parameters: breast *V*_95%_, *V*_105%,_ and hotspot, defined as dose to 1 cm^3^ (*D*_1 cc_); IMC_CTV_ *V*_80%;_ heart mean dose (*D*_mean_) and maximum point dose (*D*_max_); ipsilateral lung *V*_20 Gy_ and *V*_5 Gy_; and superficial region mean dose (*D*_mean_) and superficial region dose to 2 cm^3^ (*D*_2 cc_). Wilcoxon Signed‐Rank tests were performed between AAA and both AXB‐DM and AXB‐DW with a significance level of 0.05. To elucidate overall dosimetric trends between the two algorithms, point‐wise median dose–volume histograms (mDVH) were computed for IMC_CTV_, superficial region, and ipsilateral lung by finding the population median value in each DVH bin.

The homogeneity of the dose to the ipsilateral breast was assessed using the Homogeneity Index (HI), given by the following equation.

$$\text{HI}=\frac{D_{2\%}-D_{98\%}}{D_{\mathrm{prescribed}}}$$

**Figure 2:**
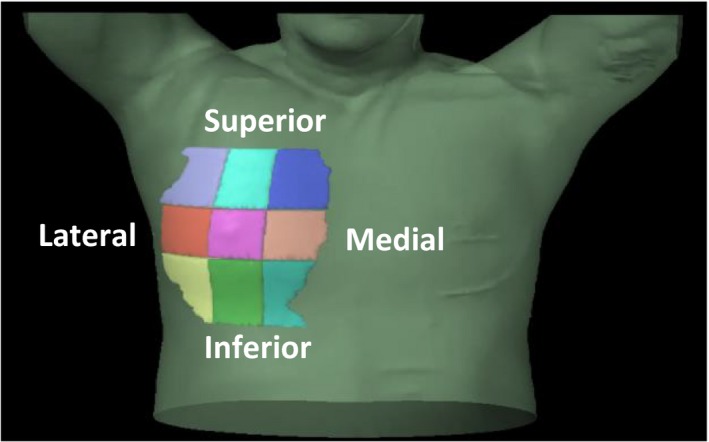
Patient body contour showing the breast divided into nine subvolume centered on the nipple. Subvolumes were created by creating a central volume of interest extending from the anterior skin to the posterior lung border centered on the nipple. Eight additional subvolumes were defined bordering on the central volume and extended to the breast contour border.

To examine possible regional trends, the breast was subdivided into nine subvolumes (Fig. 2). A central subvolume of interest in the breast was created with a side length of 4–5 cm depending on breast volume, centered on the nipple extending anteriorly to the skin and posteriorly to the lung. This central subvolume defined the center of a 3 × 3 grid of nine total subvolumes. Each volume extended from its border with the central subvolume to the border of the breast contour. The nipple was chosen as the central landmark to consistently evaluate potential high dose region in the same subvolume for each patient. A summary of the segment volumes is found in Table 2. The mean dose (*D*_mean_) and hot spot (*D*_1 cc_) was compared between the subvolumes.

**Table 2:** Breast segment volumes

	Median (cm^3^)	Range (cm^3^)
Superior medial	37.4	4.4–164.1
Mid medial	67.6	8.3–201.9
Inferior medial	37.2	1.2–136.3
Superior mid	40.4	11.3–126.6
Nipple	78.1	18.1–146.0
Inferior mid	38.8	2.0–101.1
Superior lateral	57.0	20.0–146.9
Mid lateral	131.5	17.5–248.6
Inferior lateral	40.0	0.3–156.8

To examine the effects of patient size on the difference between AXB and AAA, dose and HU profiles were collected 1 cm anterior to the 2500 cGy isodose line, which was used as an analog for the field edge. This was chosen as it represents the path of largest separation through the patient.

This study was determined to be of minimal risk and consistent with a quality improvement project using the Alberta Research Ethics Community Consensus Initiative (ARECCI) screening tool provided by the Heath Research Ethics Board of Alberta and did not require further ethics board approval.^11^

## 3 RESULTS

### 3.A Targets and OARs

**Figure 3:**
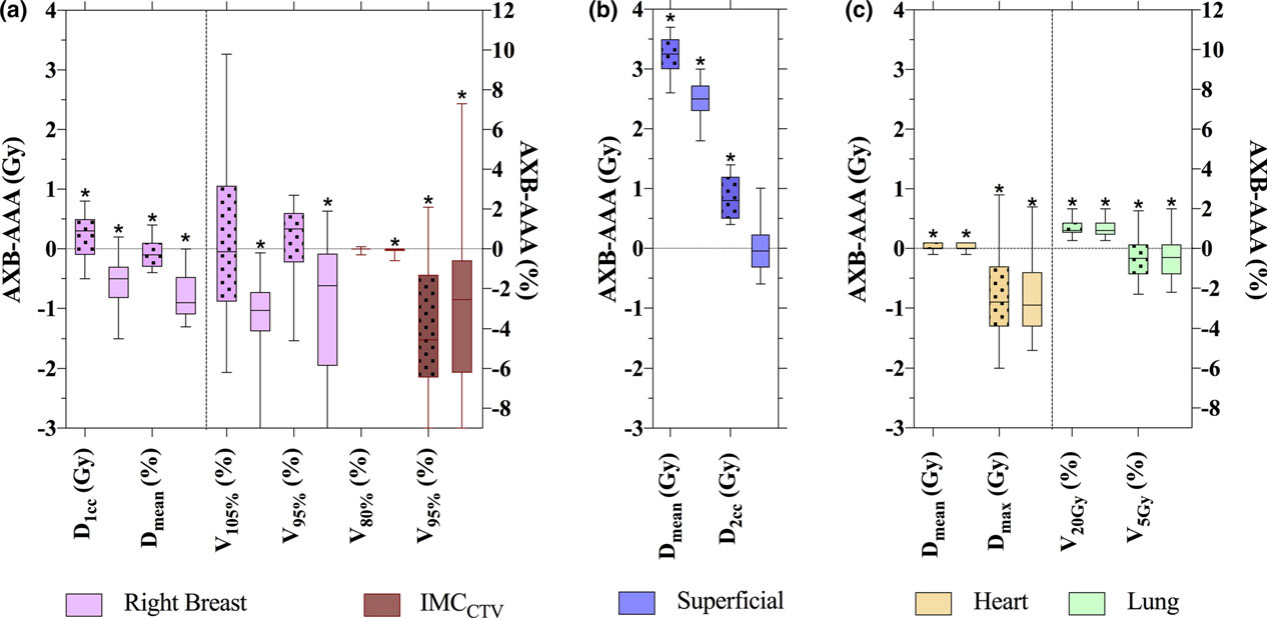
AXB–AAA differences in DVH parameters with dotted boxes indicate dose‐to‐medium, solid indicate dose‐to‐water for (a) targets: breast and IMC_CTV_ (b) superficial region, and (c) OARs: heart and ipsilateral lung. Dose parameters are measured on the left *y*‐axis, volume on the right. The whiskers indicate the range of the data and the box indicates the 25–75 percentiles. Statistically significant differences are indicated by a * above the top whisker.

Minimal differences were observed across all patients between AAA and AXB radiotherapy plans (Table 3). Figure 3(a) presents the AXB–AAA difference for targets (breast and IMC_CTV_). For AXB‐DM, the median difference for all breast metrics was less than 1 Gy or 1% and no difference was observed in homogeneity. For AXB‐DW, the *V*_95%_ coverage decreased by 2.4%, representing the maximum difference. All other breast metrics were within 1% and 1 Gy. The IMC_CTV_ showed very little change on planned target coverage (*V*_80%_); however, the *V*_95%_ had a statistically significant and potentially clinically relevant difference with almost a 5% decrease when plans were re‐calculated with both AXB‐DW and AXB‐DM.

**Table 3:** Median and range DVH parameters from both AAA and AXB calculated plans. A * indicates that the value for the parameter was significantly different on the 5% level from the value for the AAA plan

	AAA		Acuros XB dose to medium		Acuros XB dose to water	
	Median	Range	Median	Range	Median	Range
Breast						
*V*_95%_ (%)	93.6	83.1–98.3	94.9	82.0–99.4	91.2*	73.4–98.2
*V*_105%_ (%)	3.9	0.5–13.6	4.7	0.2–13.7	0.4*	0.0–7.5
Mean dose (Gy)	50.4	48.5–51.2	50.3*	48.5–51.2	49.5*	47.9–50.7
*D*_1 cc_ (Gy)	53.2	52.6–55.3	53.5*	52.6–55.2	52.6*	51.9–54.8
HI	0.2	0.1–0.9	0.1	0.1–0.9	0.1	0.1–0.9
IMCs						
*V*_80%_ (%)	100.0	99.6–100	100.0	99.3–100.0	100*	99.0–100.0
*V*_95%_ (%)	76.6	10.2–90.5	71.7*	12.3–92.2	72.6*	10.5–92.5
*D*_95%_ (Gy)	45.0	42.1–46.7	44.7*	41.9–47.0	44.5*	41.8–47.0
Lung						
*V*_20 Gy_ (%)	24.0	13.4–42.2	24.8*	14.3–43.1	24.8*	14.2–43.1
*V*_5 Gy_ (%)	47.9	33.3–64.8	46.1*	33.4–63.0	46.2*	33.5–63.1
Heart						
Mean dose (Gy)	0.8	0.6–1.3	0.9*	0.7–1.3	0.9*	0.7–1.3
Max dose (Gy)	7.3	4.7–26.2	6.2*	3.6–27.1	6.2*	3.6–26.9
Superficial region						
Mean dose (Gy)	38.2	34.3–40.9	41.5*	36.9–43.7	40.8*	36.2–42.9
*D*_2 cc_ (Gy)	51.5	49.2–52.4	52.5*	49.7–53.6	51.5	48.8–52.7

Figures 3(b) and 3(c) present the AXB–AAA difference for organ at risk (OAR) metrics. The median difference was less than 1 Gy or 1% for all OAR metrics aside from mean superficial dose. Mean superficial dose [Fig. 3(b)] showed a statistically significant difference (*P *< 0.01) with a median increase of 3.2 Gy when plans were recalculated with AXB‐DM and 2.6 Gy when recalculated with AXB‐DW.

**Figure 4:**
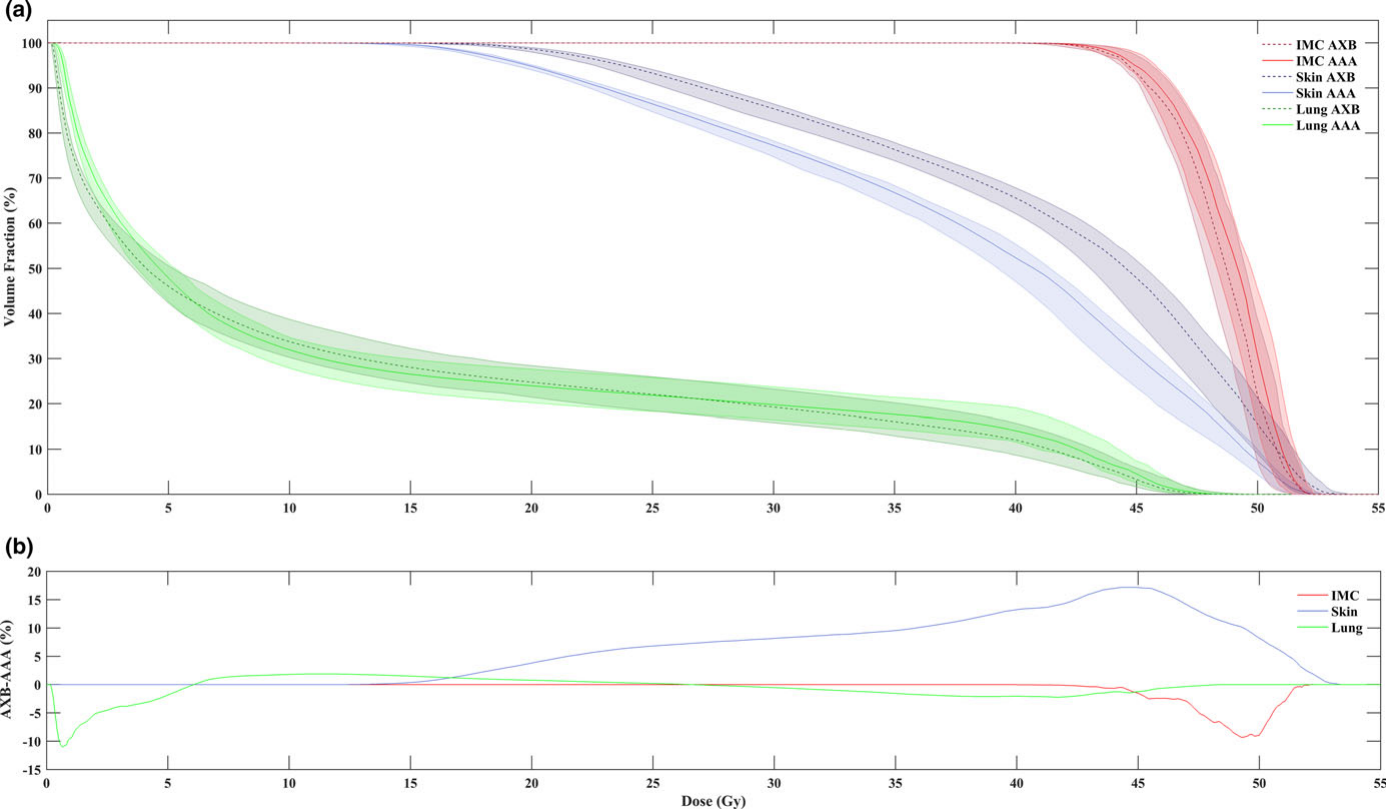
(a) Median dose–volume histograms for IMC_CTV_, skin, and ipsilateral lung. Shaded area indicates the 25–75 percentiles of the data. (b) Difference in median DVH reported as AXB–AAA (dashed minus solid line) for IMC_CTV_, skin, and ipsilateral lung. Dose‐to‐medium is shown for AXB.

Figure 4 illustrates the overall DVH trends across all patients highlighting the median and 25th and 75th percentiles in the population. The trend of minimal difference between AXB and AAA is again demonstrated for IMC_CTV_ and ipsilateral lung, while, the superficial region had consistently higher doses when calculated with AXB‐DM as observed by the separation between the curves.

### 3.B Subvolume analysis

**Figure 5:**
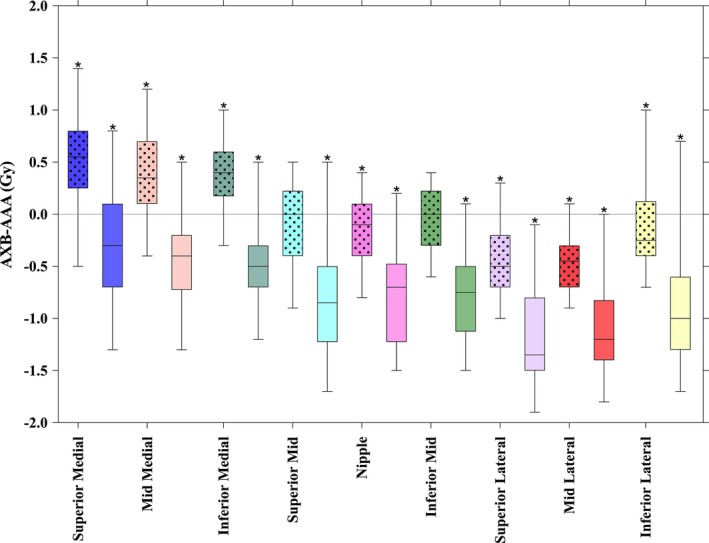
AXB–AAA differences for the breast subvolume defined in Fig. 2 with dotted boxes indicate dose‐to‐medium, solid indicate dose‐to‐water. The whiskers indicate the range of the data and the box indicates the 25–75 percentiles. Statistically significant differences are indicated by a * above the top whisker.

The results of the breast subvolume analysis are provided in Fig. 5. There was a statistically significant trend for AXB calculated plans to be slightly hotter medially and slightly cooler laterally, but all median differences were within ±1 Gy for AXB‐DM and within ±1.5 Gy for AXB‐DW. All patient‐specific differences in quadrant mean dose for both AXB‐DM and AXB‐DW were within ±2 Gy.

A hotspot analysis was performed as part of the subvolume analysis. For most patients, the hotspot was in the superior central or superior lateral subvolumes. We observed no overall trends in hotspot location between AAA and AXB.

## 4 DISCUSSION

When introducing a new dose calculation algorithm into routine patient care, it is important to have a thorough understanding of the expected differences between the old and new plans, so that clinical practice can be adjusted accordingly. This need was previously identified in the radiation oncology community while moving from 2D to 3D planning and subsequently led to the introduction of dose inhomogeneity corrections.^12^ AXB (version 10 and 11) has been extensively validated in heterogeneous phantoms against Monte Carlo (MC) and AAA calculations.^5^, ^13^, ^14^ In all cases, AXB exhibited closer agreement with MC calculations than AAA and the differences were generally less than 5%. The results of these studies support the hypothesis that AXB produces more accurate dose calculations in heterogeneous materials and has led to follow‐up clinical application studies into the implications of using AXB in lung treatment planning.^13^, ^15^, ^16^, ^17^, ^18^ The irradiation volume in whole breast radiotherapy is quite inhomogeneous with severe contour changes. The dosimetric impact of using AXB instead of AAA has been studied in standard two tangent fields with the purpose of assessing the behavior of the algorithm in different breast tissues and lung densities.^8^ Our aim was to evaluate the expected plan differences between AAA and AXB in a locoregional DIBH scenario, where the density changes are greatest, with the purpose of implementing AXB for breast planning into clinical practice.

We investigated the difference between the two AXB dose reporting modes, dose‐to‐water and dose‐to‐medium, compared to AAA. Larger magnitude differences were observed for dose‐to‐water compared to AAA than dose‐to‐medium in the breast DVH metrics. This trend is consistent with findings of Zifodoya et al.^19^ While some parameters were statistically significant, the difference between AXB and AAA plans was very small. The observed differences in breast metrics when calculating dose with AXB instead of AAA are of limited clinical relevance as median differences are all less than 5%. Similar results were found for heart, IMC_CTV_, and ipsilateral lung parameters. Thus, for commonly evaluated target volumes and OAR dose constraints and DVHs, noticeable differences between AAA and AXB are not expected during clinical implementation. The choice of dose‐to‐medium or dose‐to‐water continues to be a debate in the community.^20^, ^21^ The advantage of dose‐to‐water is that most clinical experience, including calibration and outcome data, are based on dose‐to‐water dose reporting. Dose‐to‐medium has the advantage that is the most consistent with Monte Carlo.^22^, ^23^

The largest difference between AAA and both AXB reporting modes was mean superficial dose. This is to be expected because AXB handles interfaces and inhomogeneities more accurately than AAA.^13^, ^24^ AAA has been shown to be agree more closely with MC calculations than older algorithms,^25^ though AAA is not sufficiently accurate for performing superficial dosimetry.^26^ Superficial dose is not commonly assessed using an evaluation volume; however, we have found that upon implementation of AXB for breast, physicians, and dosimetrists can expect to see higher overall superficial doses than when using AAA. Conversely, a plan originally optimized with AXB may be cooler in the superficial region than plans optimized and calculated with AAA. The 2‐cc hotspot increased for all patients with the use of AXB‐DM, but by median 1 Gy as opposed to 3.2 Gy, as was the case with the mean dose. Panettieri et al. investigated the dose in the buildup region of AAA and found that it underestimated the predictions of MC calculations severely in the first 2 mm of tissue, particularly when the beam was delivered at a wide angle as is the case in breast radiotherapy.^27^ Since AXB has been shown to agree more closely with MC calculations in the buildup region,^13^ it is consistent that it should estimate the mean dose in the superficial 5 mm to be hotter. The 2‐cc hotspot did not change with AXB‐DW.

In the subvolume analysis, there was a small but measurable trend toward higher mean dose to medial subvolumes and lower mean dose to lateral subvolumes when dose was calculated using AXB as compared to AAA. This may be a result of a combination of factors including geometric location of the prescription point in original plans, volume averaging in the subvolumes, modulation, and path through lung along chest wall interface. The contribution of each factor cannot be completely separated. As can be seen in Table 2, the lateral segments of the breast are on average larger than the medial and central segments. This could contribute to volume averaging resulting in the lower mean dose recorded in the lateral segments. Figure 1(b) shows a larger difference between AXB and AAA in the lung in the patient with the large separation compared to the patient with the smallest separation. Since AAA performs poorer near lung interfaces, it is reasonable that the largest deviations are the lateral and medial segments. The dose in these segments is most heavily influenced by the beam passing through the lung. The prescription point location is inconsistent, as it is determined as part of field‐in‐field planning process based on patient‐specific anatomy. Modulation was held as constant patient‐to‐patient as possible by having a single dosimetrist plan all patients; however, patient anatomy again is a factor in the resultant modulation.

These results may not extend to wedged field planning, especially physical wedges, where the skin dose from scatter is higher. As well, the impact on partial breast planning has not been explored. Fogliata et al.^8^ investigated the dosimetric implication of separately analyzing different tissues in the breast using AXB and AAA. This study found that AXB calculates higher dose in glandular breast tissue, which has a similar density to muscle, and does not calculate as large a difference for adipose tissue, which combined result in an overall dose to the breast that is not clinically relevant. Our results are consistent with these findings.

If large differences exist between two dose calculation algorithms, questions may arise about the impact of switching to a new dose calculation algorithm on clinical practice. For example, in lung, AXB has been shown to calculate lower dose to the edge of the tumor than AAA, which may result in physicians changing practice in order to cover the tumor to the same level as when using AAA.^13^ This behavior can be problematic when all outcomes and toxicity data are based on doses calculated using the older algorithm. Apart from superficial dose, we found no clinically relevant differences between the breast plans calculated with AAA and AXB. Upon clinical implementation of AXB for breast, clinicians should be made aware that higher superficial doses are a result of the new dose calculation algorithm.

## 5 CONCLUSIONS

In this study, the differences in dose–volume parameters and plan quality were evaluated in AXB and AAA for breast cancer. Marginal differences were found for target volume coverage and homogeneity and, aside from skin mean dose, organ at risk dose–volume parameters. As the dosimetric differences between the two algorithms are small, we conclude that using AXB for breast treatment planning would have a minimal impact on clinical practice.

## CONFLICT OF INTEREST

The authors have no conflict of interest to declare.
